# Entanglement between pharmacy/pharmaceutical education and cancer? A bibliometric answer

**DOI:** 10.2478/abm-2025-0024

**Published:** 2025-09-02

**Authors:** Siyuan Peng, Wenhao Wang, Rui Luo, Xiye Wang, Jiayue Huang, Xin Pan, Zhengwei Huang

**Affiliations:** School of Pharmaceutical Sciences, Sun Yat-sen University, Guangzhou, Guangdong510006, China; College of Pharmacy, Jinan University, Guangzhou, Guangdong 510632, China

**Keywords:** bibliometric analysis, cancer care, cancer-related research, oncology pharmacy, pharmaceutical education

## Abstract

**Background:**

The growing role of pharmacists in cancer care highlights the need to understand research trends linking pharmaceutical education and oncology.

**Objective:**

To quantitatively investigate the bibliometric status of the entanglement between pharmacy/pharmaceutical education and cancer-related issues, providing feasible information and suggestions for the developmental basis and research hotspots.

**Methods:**

Bibliometric analysis was performed using the Web of Science Core Collection. Boolean operations (BO) were used to set up the literature query set. The literature published between 1985 and 2022 was screened for the types of “article” and “review.” The citation analysis and research hotspot extraction were performed using the “analyze results” and “citation report” functions of Clarivate Analytics.

**Results:**

The bibliometric analysis of 722 relevant papers showed that the global interest in the topic is increasing and the collaboration between countries is quite active. The extraction results showed that guidelines for cancer therapy, palliative care and pain management, pharmacy staff's attitudes and knowledge, and adherence to chemotherapy were the main research hotspots and that these have been long-term discussed.

**Conclusion:**

Conducting a bibliometric study to analyze the overall publication status, developmental basis, and research hotspots will reveal the logical entanglement of the existing academic research between pharmacy/pharmaceutical education and cancer, and will provide a reference for academic development in the educational and oncological community.

Nowadays, cancer has become the leading cause of death worldwide. The Global Cancer Statistics show that the annual new cases and deaths of cancer reach 19.3 million and 10.0 million, respectively [1]. According to the Cancer Statistics 2023 in the USA, the estimated new cases and deaths of cancer exceed 1,900,000 and 600,000, respectively [2]. Inferred from these figures, cancer imposes a heavy burden on the health and economy of all societies.

Confronting the unneglectable facts about cancer, the medical community laid great emphasis on cancer management. The concept of cancer management involves, but is not limited to, prophylaxis, diagnosis, therapy, surgery, rehabilitation, and follow-up [3]. As important regions of medicine, pharmacy and pharmaceutical sciences play critical roles in laboratory discovery, industrial development, clinical trials, drug monitoring, pharmacovigilance, and cost-effectiveness of diagnostic, therapeutic, and theranostic agents. Pharmacy and pharmaceutical sciences provide strong support for the practices of cancer management [4]. Therefore, cultivating talents in the pharmacy and pharmaceutical fields is of high priority.

In this context, pharmacy and pharmaceutical education are shed light on. Pharmacy/pharmaceutical education is the foundation of talent cultivation, and the continuous revolution of the former benefits the latter. In recent decades, several studies on pharmacy/pharmaceutical education have been launched, and thousands of papers have been published. Although an effort to advance cancer management is expected, doubt arises as to what the interplay between these current studies and cancer-related issues will be like.

To address existing uncertainties, we formulated specific research questions and conducted a bibliometric analysis. The results provide objective insights into the relationship between pharmacy/pharmaceutical education and cancer-related research and aim to inform future investigations. The present study is intended to guide researchers working in this field.

Clear research questions are a prerequisite for a rigorous bibliometric study [5]. To assess the extent of overlap between pharmacy/pharmaceutical education and cancer-related topics, we established the following research questions:

1. What is the current global publication status in this area?
2. What forms the developmental foundation of this field?
3. What are the key research hotspots?

The subsequent analyses and discussions address these questions and seek to provide evidence-based answers.

## Method

**Figure 1.**
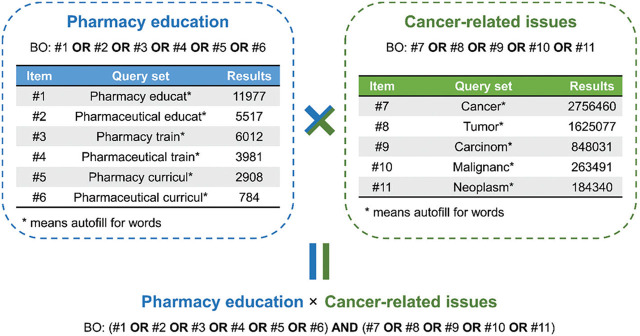
The query set with BO for the literature survey.

A bibliometric survey was conducted to address the research questions. The methodology reported in our previous study [6] was used with appropriate modification. Briefly, we consulted the Web of Science Core Collection (https://www.webofscience.com/wos), one of the most trustworthy databases of scientific papers [6], on June 29, 2023 (Beijing Time). Queries set with BO are illustrated in **Figure 1**. The query set with BO for the literature survey was BO: (Pharmacy educat* OR Pharmaceutical educat* OR Pharmacy train* OR Pharmaceutical train* OR Pharmacy curricul* OR Pharmaceutical curricul*) AND (Cancer* OR Tumor* OR Carcinom* OR Malignanc* OR Neoplasm*), and * meant autofill for words. The publication years were 1985–2022, and the publication types were “article” and “review.” The “analyze results” and “citation report” functions of Clarivate Analytics were used. To improve the credibility of the analyzed content, we would determine whether the literature in the search results is relevant to pharmacy/pharmaceutical education and cancer by skimming through the titles, abstracts, graphs, tables, and conclusions and excluding the irrelevant ones. The documents of interest were exported as TXT files (both tab-delimited and plain text), and the original document is provided in the supporting information. The exported TXT files were processed by Bibliometric Online Platform (https://bibliometric.com/) and VOSviewer software, and the resulting figures were processed by Adobe Illustrator or Photoshop for layout.

## Results

**Figure 2.**
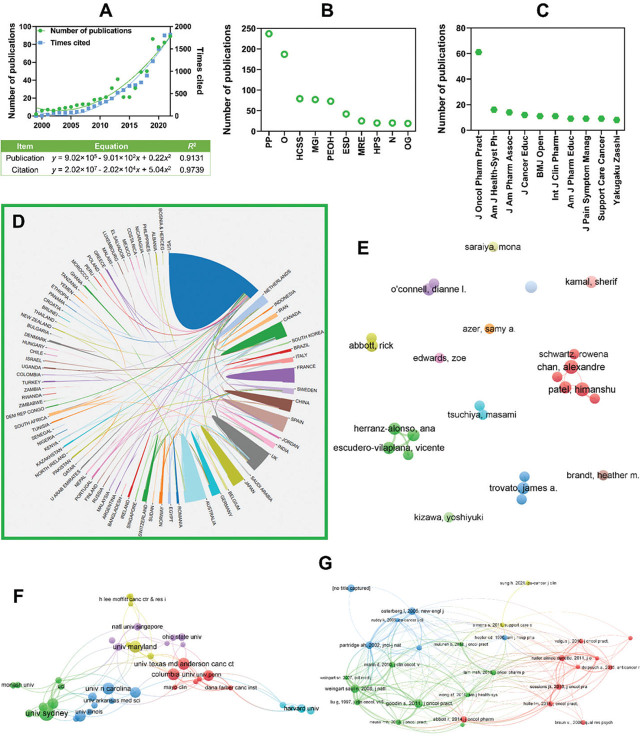
Bibliometric results-basic bibliographic information, collaboration status, and co-citation analysis. **(A)** Annual distribution of number of publications and times cited; **(B)** Top 10 research categories; **(C)** Top 10 publishing journals; **(D)** Cooperation relationship between countries; **(E)** Cooperation relationship between organizations; **(F)** Cooperation relationship between researchers; and **(G)** Clustered network of co-citation. ESD, Education, Scientific Disciplines; HCSS, Health Care Sciences & Services; HPS, Health Policy & Services; MGI, Medicine-General & Internal; MRE, Medicine-Research & Experimental; N, Nursing; O, oncology; OG, Obstetrics & Gynecology; PEOH, Public, Environmental & Occupational Health; PP, pharmacology & pharmacy.

**Figure 3.**
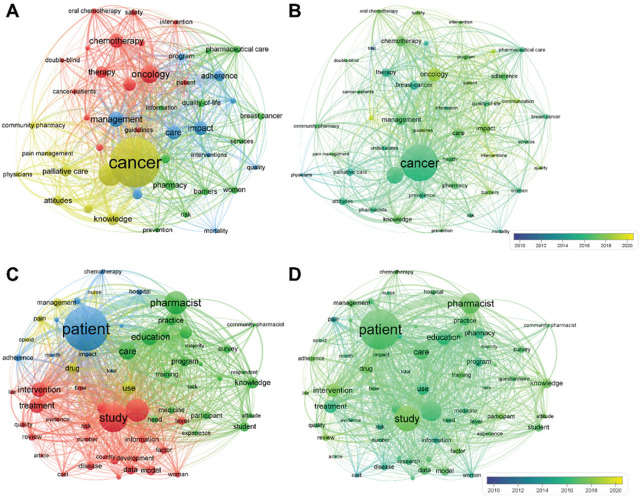
Bibliometric results-research hotspots mining. **(A)** Clustered network of keywords co-occurrence; **(B)** Time-overlay network of keywordsco-occurrence; **(C)** Clustered network of terms co-occurrence; and **(D)** Time-overlay network of terms co-occurrence.

A total of 722 documents were retrieved, and the bibliometric results are showcased in **Figures 2 and 3**. According to **Figure 2A**, since 1999, the number of publications and times cited both began to ascend demonstrated that an increasing attention was paid to this topic. The growing trends could be well fitted using a quadratic equation (*R*^2^ > 0.9), and the estimated numbers were 100 for publications and 2,000 for citations. Overwhelmingly, education only ranked sixth in research categories, and pharmacy, oncology, and healthcare were the major categories (**Figure 2B**). The top 10 publishing journals exhibit similar scenarios (**Figure 2C**), and there were 8 journals in the field of pharmacy, oncology, and healthcare, with only two journals relating to education (*J Cancer Educ* and *Am J Pharm Educ*). These results were surprising to some extent because the topic of pharmacy education was a critical component of the query set (#1–6). It was then inferred that the majority of relevant studies were designed from the perspective of pharmacy, oncology, and healthcare practices in cancer treatment, and education was partly involved.

**Figures 2D–F** shows the cooperation relationship between countries, organizations, and researchers. Overall, the status of cooperation was quite active at all levels. Organizations and researchers in the USA, Australia, UK, Canada, France, and China played important roles in academic collaborations, and both domestic and international collaborations were observed. The active collaboration was beneficial for the future development of this field.

The co-citation analysis results are depicted in **Figure 2G**. As reported in the literature, the highly co-cited documents served as the developmental basis for a certain realm [7]. Herein, we summarized the main idea of the representative documents of each cluster in **Figure 2G** to reflex the developmental basis. Red cluster: Clinical pharmacists as oncology workforce [8]. Green cluster: Recommendations for chemotherapeutic drug administration from pharmacy panel [9]. Blue cluster: Medication adherence, the broad aspect [10]. Yellow cluster: Adherence to medication in pharmaceutical care [11].

Finally, to extract the research hotspots, the co-occurrence profile of keywords and terms was analyzed. The clustered and time-overlay network was visualized in **Figure 3**. Interestingly, the co-occurrence networks of keywords and terms were “cancer”-centered (**Figure 3A**) and “patient”-centered (**Figure 3C**), respectively. The keywords and terms that were secondary to the center included “guidelines,” “palliative care,” “attitudes,” “knowledge,” “intervention,” “drug,” “adherence,” “pain,” “management,” etc. We speculated that the following three points were the main research hotspots.

(I) Guidelines for cancer therapy: Cancer is one of the diseases that seriously threaten human health. With the development of medicine and technology, a wide range of therapeutic methods to fight against cancer have emerged. But there are problems with these methods, such as whether they are effective or not, and how they should be used, and thus it is necessary for pharmacists to formulate guidelines for determining the relevant standards for treating cancer [2]. Nowadays, the World Health Organization (WHO) and national pharmacopoeias stipulate the standards of medicines for cancer treatment and also provide corresponding guidelines [1]. As countries continue to deepen their research on cancer treatment, it is believed that there will be more ways to treat cancer in the future. Of note, the new methodologies and knowledge should be passed on to more pharmacy professionals through pharmacy education. Therefore, it is necessary to pay more attention to the work of pharmacy education, so as to enrich and improve the guidelines for cancer treatment, and ultimately better guide treatment.
(II) Attitudes and knowledge of pharmacy staff: In this era of rapid advances in pharmaceutical sciences and pharmacy, pharmacy continues to intersect with other disciplines such as computer and artificial intelligence (AI) [12]. Especially interdisciplinary cooperation is becoming enriched in the investigation of drug therapy for cancer, such as computer and AI-aided drug design. Therefore, nowadays, pharmacists need to enrich not only the traditional knowledge of their own field but also other fields to ensure the effectiveness, rationality, and innovation of therapeutics. In addition, pharmacy education needs to produce pharmacists with positive and upright attitudes to ensure the authenticity, science, and feasibility of cancer-associated research. In the future, in the treatment of cancer, pharmacists need to be exploratory, positive, and non-absorbing, in addition to possessing a wealth of knowledge [13]. By this means, it will be possible to create new drugs to treat cancer and overcome the challenges of cancer in an interdisciplinary collaboration.
(III) Adherence to chemotherapy: Chemotherapy, as an important therapy for cancer treatment, has always had the problem of poor patient adherence because of its side effects and long-term therapy [11]. Solving the problem of chemotherapy adherence was, is, and will be the focus of pharmacists' research. Moreover, so many new therapies have emerged to replace chemotherapy and introduce innovation into pharmacy education [10]. For instance, the emergence of immunotherapy and other related alternatives to chemotherapy has led pharmacists to pay more attention and learn about them [14]. With the cooperation between pharmacy and other disciplines as well as the improvement of medical technology, it is believed that in the future, there will be more ways to solve the problem of chemotherapy adherence, so that cancer will no longer be a sensational disease.

It is worth mentioning that the temporal distribution of keywords and terms was relatively homogeneous (**Figures 3B,D**), indicating that these hotspots had been long-term exploited and were still provoking discussion.

## Discussion

For research question one, the overall status of global publications: The number of publications was continuously increasing, along with the citations. The studies in the field of pharmacy, oncology, and healthcare were prevailing, and education was in an auxiliary status. Collaboration was a common phenomenon, and the most productive countries were the USA, Australia, the UK, Canada, France, and China.

For research question two, the developmental basis for this field: Previous studies on clinical pharmacists as an oncology workforce, recommendations for chemotherapeutic drug administration from pharmacy panels, and medication adherence were the developmental foundation.

For research question three, the research hotspots: Guidelines for cancer therapy, palliative care, and pain management, pharmacy staff's attitudes and knowledge, and adherence to chemotherapy were the long-term research hotspots in this field.

With in-depth interpretation, we might come to an answer to the question of what the interaction between pharmacy/pharmaceutical education and cancer is like. On one hand, pharmacy/pharmaceutical education was involved in cancer management, but played an auxiliary part. On the other hand, investigators in clinical pharmacy paid more attention to cancer-related issues than other subareas of pharmacy or pharmaceutical sciences.

The above results and discussions offered useful insights for understanding the entanglement between pharmacy/pharmaceutical education and cancer-related issues. Currently, few publications are scrutinizing such a topic. We believed that the following information revealed in the present study would attract the attention of investigators, educators, policymakers, and also oncologists.

Despite this, it should be noted that the present study has some limitations, particularly as the consulted database was limited to the Web of Science Core Collection. As a result, some valuable documents not indexed in this database may have been omitted. In future studies, we plan to include additional databases such as PubMed, Scopus, and Google Scholar to ensure a more comprehensive literature coverage [15]. Our search was limited to English-language publications; however, significant efforts in pharmacy development and cancer research are being made worldwide, and thus, relevant literature on the intersection of pharmacy/pharmaceutical education and cancer in other languages may have been excluded. Our literature processing and measurement methods can also be further optimized, for example, with the help of other established tools such as R language, Citespace, etc., to further assist in the bibliometric process [16, 17]. Furthermore, with the rapid advancement of AI, an increasing number of researchers are employing AI to collect and analyze large datasets. We believe that the integration of generative AI and machine learning tools in large database analyses offers unique advantages, enabling more meaningful and in-depth data interpretation [12]. Nevertheless, the current findings provide sufficient insight to stimulate interest and promote progress in this field.

In conclusion, we anticipate that our analyses will serve as a valuable resource for researchers seeking to understand and investigate the intersection between pharmacy/pharmaceutical education and cancer. We also encourage greater scholarly engagement in this field. Identifying research hotspots within this intersection has the potential to enhance pharmacy education frameworks and contribute novel insights to cancer treatment strategies.

Sustained efforts to strengthen interdisciplinary collaboration will broaden the scope of the discipline, fostering innovation in pharmacy research. The integration of advanced analytical tools will enhance the scientific rigor and reliability of experimental findings, ultimately facilitating the development of novel therapeutic approaches to cancer.

Emphasizing the role of pharmacy education in cancer care is essential for training competent, motivated, and reliable pharmacists who can support researchers in addressing the complex challenges posed by cancer to human health. Through these endeavors, the relationship between pharmacy/pharmaceutical sciences and oncology will be further consolidated, leading to improved standards of cancer treatment. This will, in turn, provide more effective guidance for future pharmacy research and promote the advancement of critical research areas, including cancer therapy.

## Conclusion

The present study conducted a comprehensive bibliometric analysis to elucidate the interaction between pharmacy/pharmaceutical education and cancer. Key aspects, including the overall publication status, foundational developments, and emerging research hotspots, were systematically identified and examined. We anticipate that these findings will serve as a valuable reference for both the educational and oncological communities. Furthermore, we recommend that leading journals maintain a sustained focus on this important topic to encourage and attract high-quality research contributions.
